# Herboxidiene triggers splicing repression and abiotic stress responses in plants

**DOI:** 10.1186/s12864-017-3656-z

**Published:** 2017-03-27

**Authors:** Sahar AlShareef, Yu Ling, Haroon Butt, Kiruthiga G. Mariappan, Moussa Benhamed, Magdy M. Mahfouz

**Affiliations:** Laboratory for Genome Engineering, Division of Biological Sciences, 4700 King Abdullah University of Science and Technology, Thuwal, 23955-6900 Saudi Arabia

**Keywords:** Alternative splicing, GEX1A, Pladienolide B, Splicing inhibitors, SR proteins, Abiotic stress responses, ABA

## Abstract

**Background:**

Constitutive and alternative splicing of pre-mRNAs from multiexonic genes controls the diversity of the proteome; these precisely regulated processes also fine-tune responses to cues related to growth, development, and stresses. Small-molecule inhibitors that perturb splicing provide invaluable tools for use as chemical probes to uncover the molecular underpinnings of splicing regulation and as potential anticancer compounds.

**Results:**

Here, we show that herboxidiene (GEX1A) inhibits both constitutive and alternative splicing. Moreover, GEX1A activates genome-wide transcriptional patterns involved in abiotic stress responses in plants. GEX1A treatment -activated ABA-inducible promoters, and led to stomatal closure. Interestingly, GEX1A and pladienolide B (PB) elicited similar cellular changes, including alterations in the patterns of transcription and splicing, suggesting that these compounds might target the same spliceosome complex in plant cells.

**Conclusions:**

Our study establishes GEX1A as a potent splicing inhibitor in plants that can be used to probe the assembly, dynamics, and molecular functions of the spliceosome and to study the interplay between splicing stress and abiotic stresses, as well as having potential biotechnological applications.

**Electronic supplementary material:**

The online version of this article (doi:10.1186/s12864-017-3656-z) contains supplementary material, which is available to authorized users.

## Background

Eukaryotes use transcriptional and post-transcriptional regulatory mechanisms to respond and adapt to their environment [1]. Given their sessile nature and ever-changing environmental conditions, plants exhibit strong plasticity at the epigenome and transcriptome levels to continuously adapt to a variety of growth and stress cues [1–4]. Plants employ intricate molecular regulatory mechanisms to produce the correct transcriptome and proteome patterns to ensure survival and successful completion of their life cycles [4]. Pre-mRNA splicing is an essential post-transcriptional mechanism that removes intronic sequences from the pre-mRNA to generate mature transcripts, enabling the correct protein to be produced [5, 6]. Different co- and post-transcriptional processes are subject to sophisticated regulatory mechanisms during pre-mRNA capping, splicing, polyadenylation, export, stability, and translation [7]. Therefore, pre-mRNA splicing is regulated at many levels; also, *cis* and *trans* factors regulate the splicing and maturation of pre-mRNA and the functioning of the spliceosome.

Splicing is carried out by the spliceosome, an extremely sophisticated, dynamic macromolecular machine composed of RNAs, protein complexes, and sub-complexes that mediate a variety of RNA-RNA, RNA-protein, and protein-protein interactions [5, 8]. The spliceosome, a megaDalton ribonucleoprotein complex, comprises five ribonucleoprotein sub-complexes (snRNPs: U1, U2, U4, U5, U6) and more than 200 associated proteins [9]. The splicing machinery recognizes *cis*-regulatory elements in the pre-mRNA, leading to the assembly or disassembly of spliceosome sub-complexes. Such *cis*-regulatory elements are key for spliceosome assembly and the recruitment of *trans*-acting factors that help it function [4, 5, 10, 11]. The spliceosome machinery uses a variety of sequence information and signal inputs in the pre-mRNA to assemble and execute the splicing process, leading to the production of mature mRNAs. Such pre-mRNA *cis*-regulatory elements include the 5′ splice site (5′SS), 3′SS, branch point sequence (BPS), and polypyrimidine tract. Identification of the correct signal sequences by spliceosome subunits is essential for producing accurately spliced transcripts and, subsequently, correct proteins.

Alternative splicing (AS) involves the production of more than one mRNA isoform from the same gene through the use of alternative 5′SS, 3′SS, or both, as well as through intron retention (IR) [12]. In mammals, exon skipping constitutes the majority of splicing events. However, in plants, IR produces the vast majority of AS isoforms [4]. The use of cryptic splice sites leads to the generation of splicing isoforms that may or may not have cellular functions. Skipping strong splice signals or the recognition of weak splice signals leads to the formation of different mRNA isoforms [1, 6, 13]. Therefore, AS expands and increases the diversity of the proteome. AS is regulated in a cell-type and tissue-specific manner, as well as at different developmental stages and in response to growth, developmental, and biotic/abiotic stress conditions [13]. AS events play key roles in various abiotic stress responses in plants [7]. Recent studies have shown that more than 60% of intron-containing genes produce different splicing variants [14, 15]. Interestingly, most transcripts produced in plants via IR contain premature stop codons [15]. One important issue is understanding the fate and molecular roles of IR transcripts and their interplay with stress signals. These IR transcripts might play functional roles in the cell by titrating out functional transcript isoforms. It is also possible that IR-produced transcripts are exported to the cytoplasm and translated to generate functional peptides or small proteins. Furthermore, such IR transcripts could localize to the nucleus in unprocessed form and, after the cessation of stress cues, they could be immediately exported to the cytoplasm and translated.

Molecular studies of splicing in plants have been hampered by the lack of an *in vitro* splicing system [1]. However, RNA-sequencing (RNA-seq) studies of various model and non-model plants have produced vast amounts of data, which have greatly advanced the pace and depth of our understanding of splicing regulation and its response to various signals [16]. A variety of microbial metabolites capable of perturbing the splicing machinery have been identified and shown to exhibit cytotoxic effects in cancer cells [17, 18]. These small molecules affect alternative and constitutive splicing through targeting of the U2snRNP complex [19]. Given the conservation of the splicing machinery across eukaryotes, plant systems could be used to identify and characterize splicing inhibitors derived from natural and synthetic sources with great potential for use in mammalian cells for basic research and as therapeutic compounds.

Probing the functions of the splicing machinery and its regulation, and subsequently the interplay between these regulatory mechanism and stress signal inputs, requires the availability of small molecules capable of perturbing the splicing machinery in a targeted fashion [10, 17, 20, 21]. The use of such small molecule inhibitors in plant research would provide mechanistic insights into the splicing process and its intricate regulatory mechanism at various molecular levels and under a variety of growth and stress conditions. Work in cultured mammalian cells has identified a group of splicing inhibitors, including PB, SSA and GEX1A [20, 22, 23]. Interestingly, such compounds have distinct and very different chemical structures, and target SF3B, a subcomplex of the U2 snRNP spliceosomal complex composed of SF3B1, SF3B2, S3B3, SF3B4, SF3B5, and SF3B6, subsequently disrupting the early stages of spliceosome assembly and impairing splicing functions. The use of these compounds results in cell arrest at the G1/G2/M phase. Detailed studies have shown that these compounds bind SF3B1 in a non-covalent manner, subsequently impairing the splicing process [23].

GEX1A (herboxidiene), a compound isolated from *Streptomyces sp.* cultures, exhibits antitumor activity by targeting the spliceosome U2 snRNP complex and inhibiting pre-mRNA splicing, this activity makes GEX1A a valuable starting point for the development of anticancer drugs [20, 24, 25]. Preliminary studies have shown that GEX1A functions as an herbicide, but the mode of action is currently unknown [18, 23]. In this study, we identified GEX1A as a splicing modulator capable of perturbing constitutive and alternative splicing in plants. GEX1A triggered abiotic stress responses and ABA signaling in plants. Splicing stress signaling generated by GEX1A treatment is differentially regulated to ensure plant adaptation to stress conditions. Therefore, GEX1A can be used to probe the functions of the splicing machinery and the dynamic regulation of such machinery in response to stress conditions. Our study highlights the suitability of plant systems for screening and identifying splicing inhibitors and investigating the splicing machinery and its regulation during responses to stress factors across eukaryotic species.

## Results

### GEX1A inhibits plant growth and development and affects the splicing efficiency of a set of genes

**Fig. 1:**
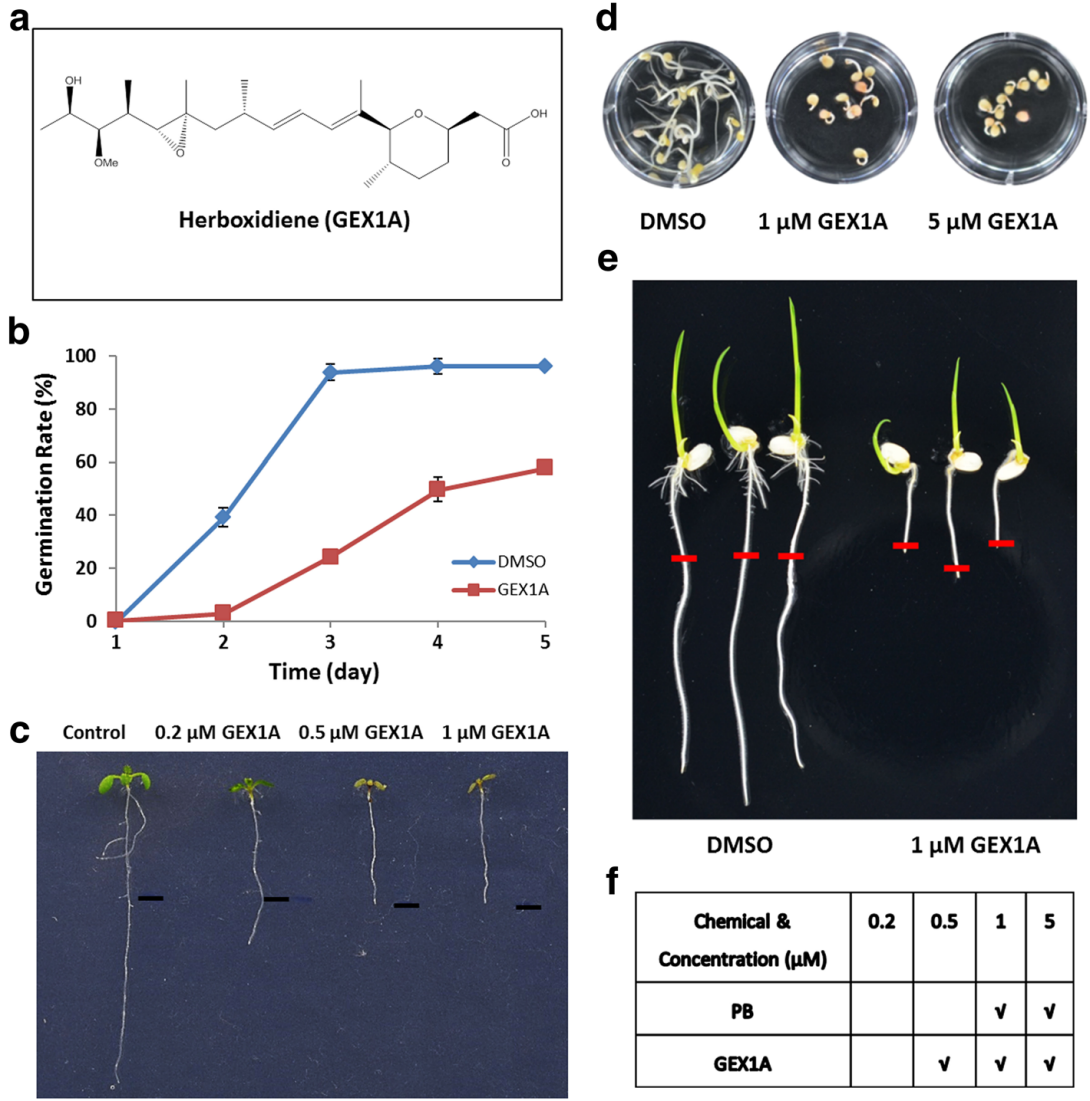
GEX1A inhibited plants growth and development. **a**, the structure of GEX1A (herboxidiene). **b**, Effects of GEX1A on Arabidopsis seed germination. GEX1A inhibits seed germination in a dose-dependent manner. **c**, Inhibition of primary root elongation of Arabidopsis seedlings by GEX1A. 5-day-old Col-0 seedlings transferred from MS medium containing DMSO (control), 0.2 μM, 0.5 μM, and 1 μM GEX1A for an additional 4 days. **d**, GEX1A inhibits tomato seeds germination. Tomato seeds was incubated in water with DMSO (negative control), 1 μM and 5 μM GEX1A for 8 days. **e**, GEX1A inhibits rice root elongation. The rice seeds were germinated on ½ MS plate for 3 days, then transfer onto ½ MS with 1 μM GEX1A for 2 days. The *red bar* marks root tip of the transferring time. **f**, comparison inhibition effect of PB and GEX1A on Arabidopsis root growth, “√” stands for 0% root elongation rate on the chemical

Very recently, we demonstrated that the macrolide pladienolide B (PB) causes global repression of pre-mRNA splicing [26]. The resulting splicing stress strongly inhibits plant growth and development. These findings encouraged us to identify more splicing inhibitors and modulators in plants for a variety of applications. Since PB, GEX1A, and spliceostatin A (SSA) function as splicing inhibitors in mammalian cells [17, 23, 27], we tested the effects of both GEX1A and SSA on plant growth and development. Whereas SSA did not have substantial effects on Arabidopsis growth and development [26], GEX1A had substantial effects on these processes. For example, GEX1A significantly delayed Arabidopsis seed germination. All viable seeds germinated on control medium at 3 days after sowing (DAS), whereas less than 25% of seeds germinated on medium supplemented with 5 μM GEX1A. GEX1A also inhibited Arabidopsis seedling growth and development: 5-day-old seedlings exhibited much shorter primary and lateral roots and smaller aboveground parts after transfer to medium containing 0.2 μM GEX1A for an additional 4 days compared to the control. Seedlings transferred to MS medium supplemented with 0.5 or 1 μM GEX1A ceased growth, and neither well-developed true leaves nor root elongation were detected (Fig. 1). To rule out the possibility that this effect is specific to one plant species, we applied GEX1A to rice (*Oryza sativa*) and tomato (*Solanum lycopersicum*), resulting in the cessation of growth and development (Fig. 1). Furthermore, we compared the effects of GEX1A and PB on plant growth and development and found that GEX1A had more potent effects on these processes than PB. For example, in a dose-response assay, 0.5 μM GEX1A had substantial inhibitory effects comparable to those of 1 μM PB (Fig. 1).

**Fig. 2:**
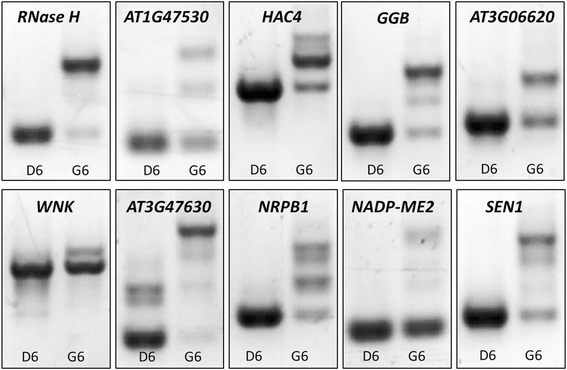
GEX1A alters the splicing patterns of a set of genes. The cDNAs were prepared from one-week-old Arabidopsis seedlings treated with GEX1A (5 μM) for 6 h, with DMSO as control. RT-PCR was performed using primers that flank introns, the gene name/locus identifier is shown. D6, DMSO 6 h, G6, GEX1A 6 h

PB, SSA, and GEX1A target the SF3b1 sub-complex of the U2 snRNA complex [27]. We recently showed that PB induces transcriptional patterns similar to those induced by a variety of abiotic stresses, and intriguingly, PB induces significant repression of splicing along with significant levels of intron retention [26]. To investigate whether GEX1A triggers similar responses, we tested its effects on the splicing of a select group of genes that are alternatively spliced under unfavorable growth conditions [28–30]. For these genes, GEX1A treatment resulted in reduced splicing efficiency, leading to splicing repression with IR (Fig. 2). For example, the gene encoding RNase H underwent strong splicing repression and lost almost all of its constitutive splicing isoforms via significant intron retention. However, *WNK* and *NADP-ME2* produced constitutive splicing isoforms, and the production of IR isoforms resulted from GEX1A-induced splicing stress. Thus, our results demonstrate that GEX1A reduces pre-mRNA splicing efficiency for a set of genes in Arabidopsis, revealing its effects on the splicing machinery.

### GEX1A produces gene expression patterns similar to those induced by a variety of abiotic stresses

Since GEX1A inhibits plant growth and development and perturbs the splicing of a set of genes, we attempted to understand the molecular functions of GEX1A by investigating its genome-wide effects on transcript levels. We performed RNA-seq of control versus GEX1A-treated plants, finding that as more reads were generated, the number of newly discovered genes plateaued, indicating that the sequencing reached saturation and we had extensive sequencing coverage (Additional file 1: Figure S1). Moreover, in a plot comparing Fragments Per Kilobase of transcript per Million mapped reads (FPKM) gene expression values between the two replicates, the differences appear to be narrow and distributed along a central line (Additional file 1: Figure S1). Clustering of gene expression levels between the control and GEX1A treatments demonstrated good consistency between the two replicates (Additional file 1: Figure S1). These results indicate that the quality of sample collection and RNA-seq was good. Our data show that 408 genes were downregulated and 561 genes were upregulated by GEX1A treatment (Additional file 1: Figure S1).

**Fig. 3:**
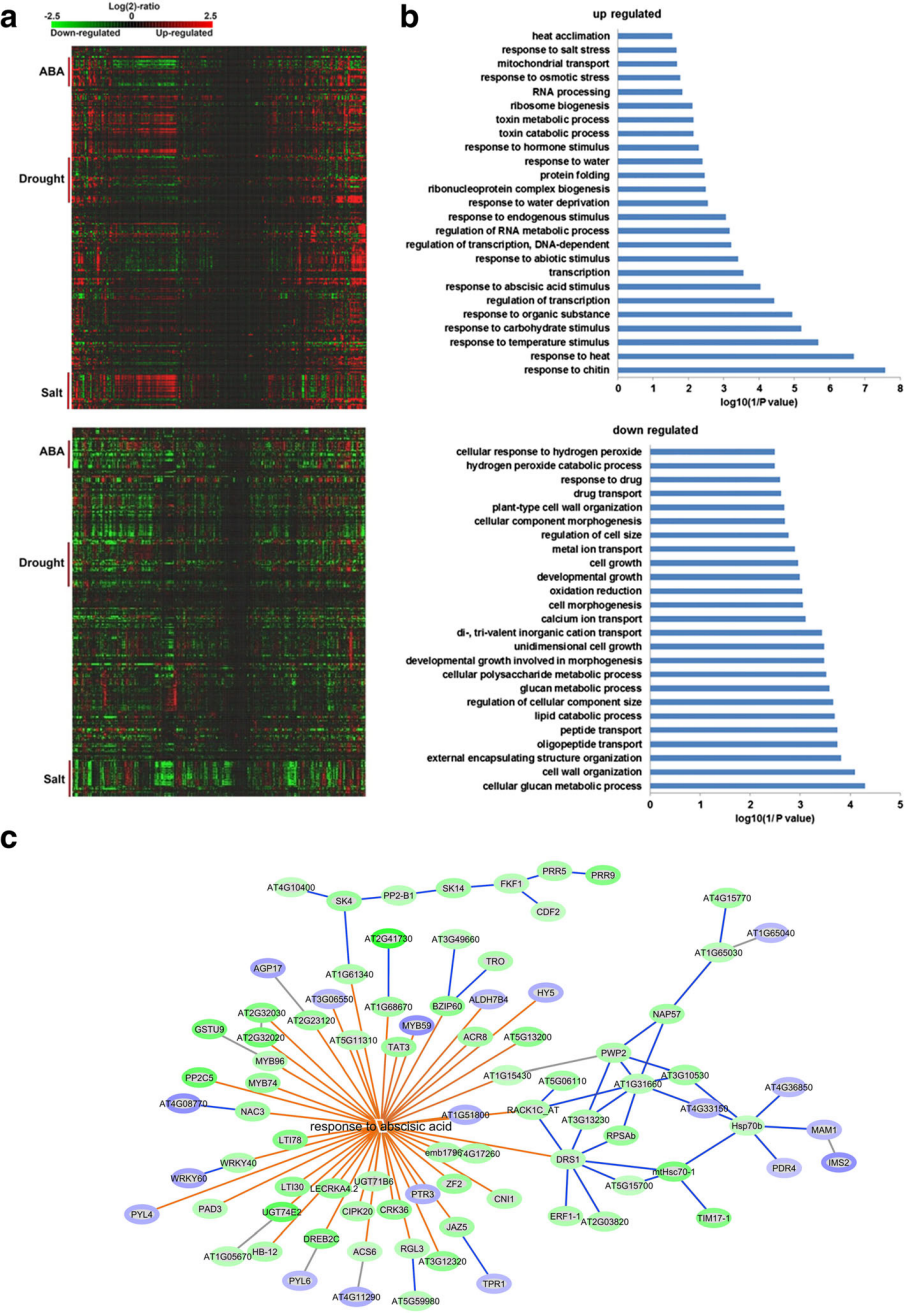
Gene expression changed by GEX1A corresponding to stress responses. **a**, upper panel, a heatmap was generated by mapping 400 randomly chosen upregulated genes in GEX1A treatment to the microarray database using Genevestigator. The heatmap indicates that a great number of these genes are upregulated (*red*) by ABA, drought and salt stress. Bottom panel: a heatmap was generated by mapping 400 randomly chosen down-regulated genes in the GEX1A treatment to the microarray database using Genevestigator. The heatmap indicates that a great number of these genes are downregulated (*green*) by ABA, drought, and salt stress. **b**, Functional categorization of regulated genes. Functional categorization (biological process) of up/downregulated genes in GEX1A treatment. Top 25 enriched pathways are shown. **c**, Differentially expressed genes in GEX1A treatment were mapped onto the response-to-abscisic-acid pathway. The analysis was performed using the Exploratory Gene Association Networks (EGAN) software tool. *Orange lines* show the participation of the genes in abscisic acid-activated signaling pathways and *blue lines* show known interactions between the genes connected. *Green ovals* represent upregulated genes and *blue ovals* represent downregulated genes

**Fig. 4:**
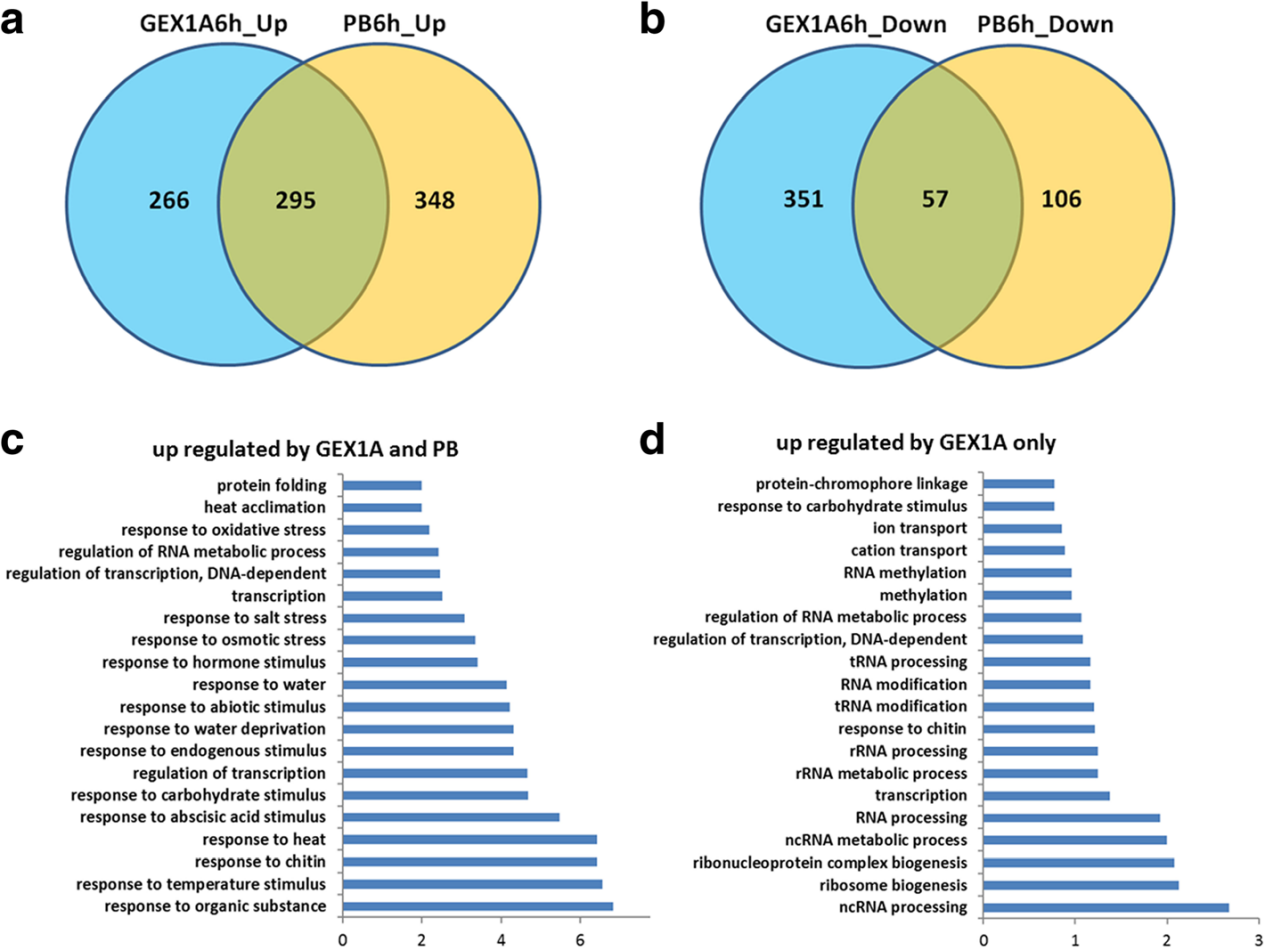
Comparison of differentially expressed genes in response to GEX1A and PB. **a**, Venn diagram showing a comparison of the upregulated genes identified in 6 h GEX1A treatment and 6 h PB treatment. **b**, Venn diagram showing a comparison of the downregulated genes identified in 6 h GEX1A treatment and 6 h PB treatment. **c**, Functional categorization (biological process) of upregulated genes in both GEX1A and PB treatments. The top 20 enriched pathways are shown. **d**, Functional categorization (biological process) of upregulated genes in only GEX1A treatment. The top 20 enriched pathways are shown

Interestingly, by mapping 400 randomly selected downregulated genes in the GEX1A treatment group to a microarray database using Genevestigator, we found that many of these genes are upregulated by a variety of abiotic stresses, including salt, drought, and ABA treatment (Fig. 3, Additional file 1: Figure S2). Similarly, many upregulated genes in the GEX1A treatment group are also induced by salt, drought, and ABA, suggesting that GEX1A triggers abiotic stress-like transcriptional patterns (Fig. 3, Additional file 1: Figure S2). Intriguingly, the genes that were differentially expressed under GEX1A treatment mapped onto the ABA signaling pathway, as revealed using Exploratory Gene Association Networks (EGAN) software (Fig. 3). These results suggest that GEX1A activates abiotic stress response genes, and they implicate ABA signaling in plant responses to GEX1A treatment. Next, we compared the effects of PB and GEX1A on the transcription patterns of these genes. Interestingly, at 6 h of treatment, nearly 50% (295) of the upregulated genes were upregulated by both GEX1A and PB. Moreover, nearly 50% of the genes downregulated by PB were also downregulated by GEX1A treatment. Therefore, GEX1A and PB induce similar transcriptional patterns (Fig. 4). Interestingly, functional categorization of the upregulated genes by GEX1A and PB relate to abiotic stresses whereas those upregulated by GEX1A only relate to RNA processing. These data indicate the interplay between the splicing stress and abiotic stresses.

### GEX1A activates abiotic stress- and ABA-inducible promoters and modulates stomatal aperture

**Fig. 5:**
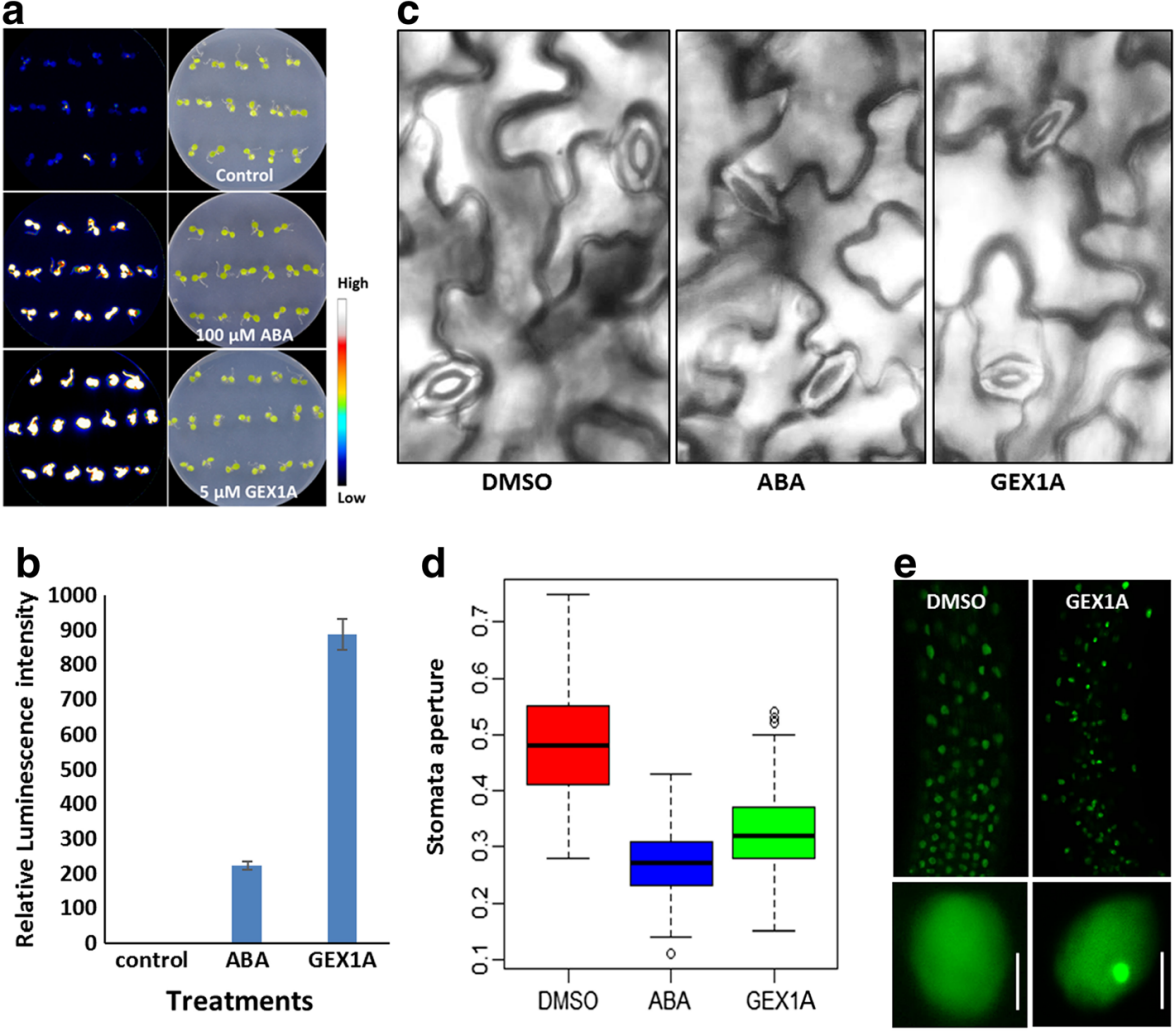
GEX1A treatment induced RD29A-LUC expression and stomatal aperture closure, caused relocation of SR45 proteins. **a**, One-week-old *RD29A-LUC* transgenic seedlings were treated with DMSO, 100 μM ABA or 5 μM GEX1A for 6 h, then sprayed with D-luciferase and observed by CCD camera. **b**, Relative bioluminescence intensities of RD29A-LUC seedlings in each treatment. **c**, Leaves of 3–4-week-old Arabidopsis plants were treated in opening solution for approximately 2.5 h and then transferred into opening solution with 20 μM GEX1A for 4 h. DMSO and ABA were used as negative and positive controls, respectively. **d**, Boxplot comparison of stomatal aperture in Arabidopsis leaves after the indicated treatments, three replicates and 150 stomata were measured. **e**, One-week-old *35S::SR45:GFP* transgenic seedlings were treated with DMSO (left) or 5 μM GEX1A (right) for 24 h. Left-upper, GFP signal in the elongation zone of a *35S::SR45:GFP* root in control conditions. Left-bottom, close-up view of nuclei of elongation zone cells from DMSO-treated *35S:SR45:GFP* transgenic plants. Right-upper, GFP signal in the elongation zone of a *35S::SR45:GFP* root in GEX1A treatment. Right-bottom, close-up view of nuclei of elongation zone cells from 5 μM GEX1A-treated *35S::SR45:GFP* transgenic plants, nuclear speckles formed in the nuclei

Because our data on the effects of GEX1A treatment on genome-wide transcriptional patterns indicate that GEX1A activates abiotic stress response genes and maps onto the ABA pathway, we investigated whether GEX1A treatment activates abiotic stress- and ABA-inducible promoters using *RD29A::LUC* Arabidopsis plants; the *RD29A* promoter is induced by a variety of abiotic stresses, including salt, cold, drought, and ABA [31]. The *RD29A* promoter was significantly induced by GEX1A treatment, corroborating our genome-wide expression data (Fig. 5). Furthermore, we used another stress-inducible promoter, the *MAPKKK18* promoter, to drive the *uidA* gene (*MAPKKK18::uidA*) [32]. Similarly, the *MAPKKK18* promoter was induced by GEX1A treatment (Additional file 1: Figure S2). These data indicate that GEX1A treatment induces abiotic stress- and ABA-responsive genes.

Next, we investigated whether GEX1A triggers other physiological responses similar to those of ABA by testing the effect of GEX1A on stomatal aperture. GEX1A treatment led to a reduction in stomatal aperture similar to the effect of ABA, suggesting that GEX1A triggers the ABA signaling pathway (Fig. 5). Because the *sr45* mutant is hypersensitive to ABA treatment, we investigated whether GEX1A would affect the sub-nuclear localization of SR45:GFP. Interestingly, GEX1A treatment led to the formation of larger nuclear speckles compared to the control (Fig. 5), indicating that the inhibition of splicing leads to the accumulation of SR proteins and likely other splicing factors. It should be noted that ABA treatments did not induce the formation and sub-nuclear localization of SR45-GFP.

### GEX1A significantly inhibits pre-mRNA splicing

**Fig. 6:**
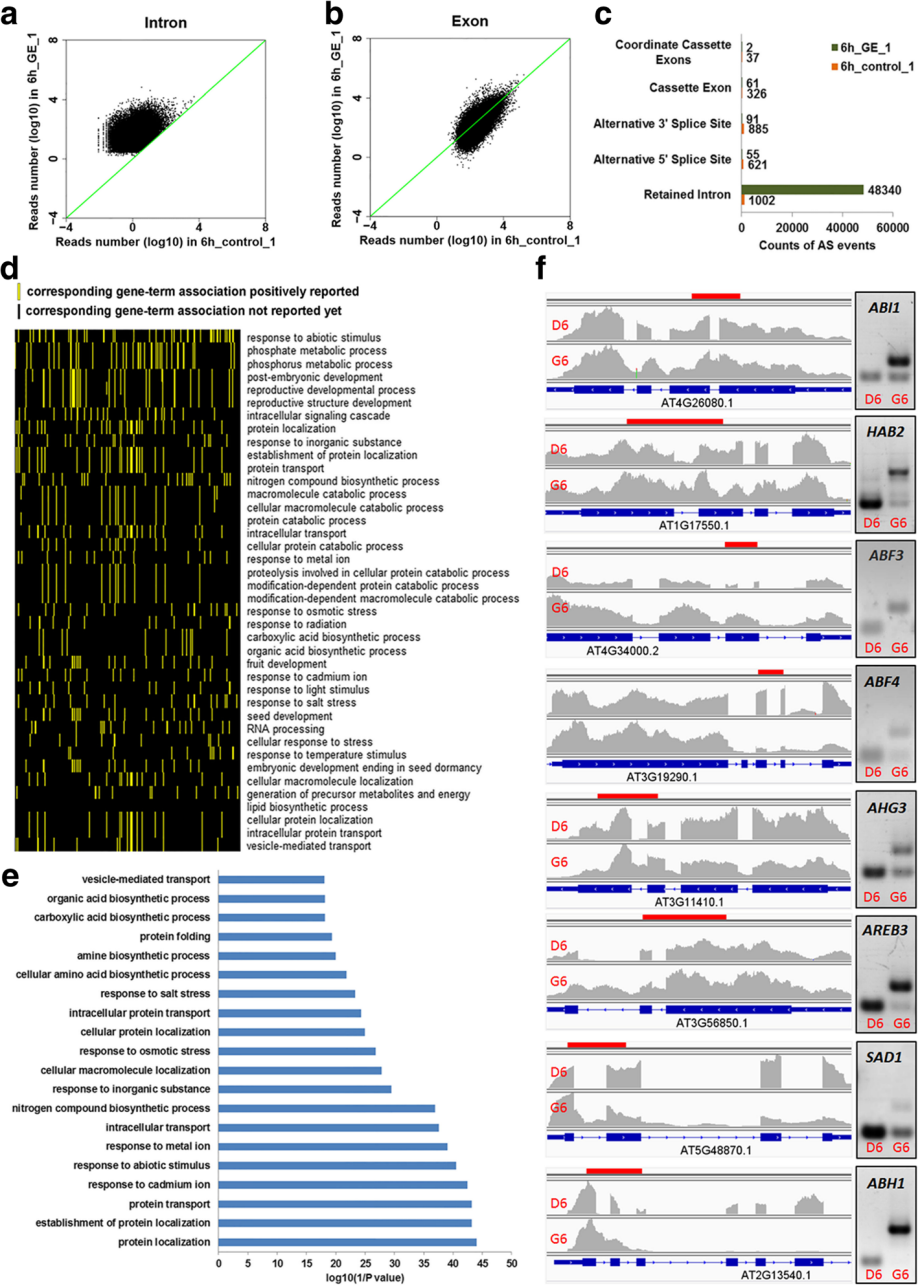
Genes with perturbed splicing in GEX1A treatment are associated with stress responses. **a** and **b**, Comparison of intron retention between control and GEX1A treatments. Reads numbers for the exons and introns are plotted. The expression of introns (**a**), but not exons (**b**), in GEX1A treatments showed a global upregulation. **c**, Comparison of global alternative splicing between control and GEX1A treatments. The intron retention events increased in the drug-treated samples, while the other AS events (including alternative 5’ splice sites, 3’ splice sites, and exon skipping) decreased in the GEX1A-treated samples. **d**, A two-dimensional view of the functional annotations of genes with retained introns in GEX1A treatment. The functional classification of genes was done using the DAVID software. The top 40 functional annotations were ordered by the number of genes in each category and selected for the two-dimensional view, which indicates that genes with retained introns were strikingly enriched in the response-to-abiotic-stress category. **e**, Functional category (biological process) of genes with retained introns in GEX1A treatment. The top 20 categories were ordered by the enrichment scores and selected. **f**, RT-PCR. The cDNAs were prepared from one-week-old Arabidopsis seedlings treated with 5 μM GEX1A for 6 h, DMSO as control. Gene structure and intron retention of interesting regions from eight genes were shown in IGV program, validation of intron retention of each gene was performed by RT-PCR using intron-flanking primers, with the result shown on the right. The *red bar* in the IGV program snapshot represents the target amplification region. D6, DMSO 6-h treatment; G6, GEX1A 6-h treatment

Next, we investigated whether GEX1A triggers genome-wide of repression splicing, similar to the effect of PB. We therefore used the same experimental conditions that were previously used with PB and the same pipelines and parameters used for data analysis of pre-mRNA splicing events [26, 33] to study the genome-wide effects of GEX1A on pre-mRNA splicing and to compare the effects of GEX1A with those of PB. In the control samples, 97% of sequenced reads mapped to exons, 1% mapped to introns, and the remaining 2% mapped to intergenic regions. By contrast, in the GEX1A-treated samples, approximately 89–90% of reads mapped to exons, and the percentage of intron reads was significantly higher (8–9%), while the percentage of intergenic region reads was the same as that of the control (Additional file 1: Figure S3). By plotting the expression intensity of introns and exons between GEX1A treated and control samples, we found that the expression of introns, but not exons, in GEX1A treated samples was globally upregulated (Fig. 6).

GEX1A treatment led to significant perturbation of splicing, including 48,340 IR events in the GEX1A 6 h treatment group compared to only 1002 in the DMSO control. Interestingly, the frequency of other forms of AS was significantly reduced. For example, the DMSO control exhibited 621 alternative 5′SS events compared to only 55 events in the GEX1A treatment group. The DMSO group exhibited 885 alternative 3′SS events compared to only 91 events in the GEX1A treatment group. There were 326 cassette exon (exon skipping) events in the DMSO control compared to only 61 in the GEX1A treatment group. Finally, there were 37 coordinate cassette exon events in the DMSO control compared to only two events in the GEX1A treatment group (Fig. 6). Furthermore, functional categorization of genes, biological processes, with retained introns in GEX1A treatment reveal that these genes belong to abiotic stress responses, protein transport and RNA processing (Fig. 6). We validated some of the intron retention events of these genes using RT-qPCR (Fig. 6). These data indicate that GEX1A perturbs the splicing machinery, leading to splicing inhibition and the significant accumulation of IR events.

**Fig. 7:**
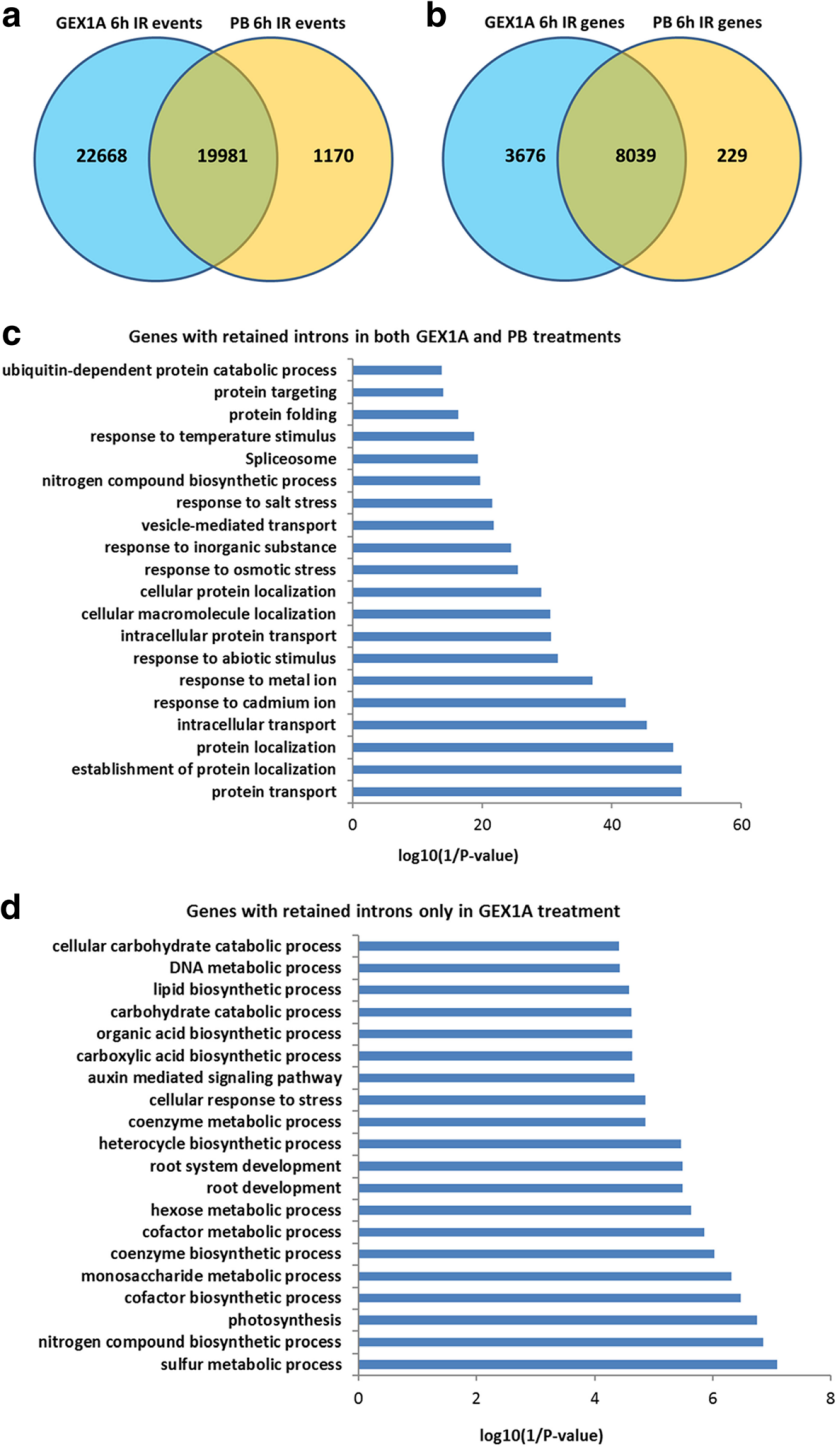
Comparison of intron-retention events and genes between GEX1A and PB treatments. **a**, Venn diagram showing a comparison of the intron-retention events identified in 6-h GEX1A treatment and 6-h PB-treatment. **b**, Venn diagram showing a comparison of the intron retention genes identified in 6-h GEX1A treatment and 6-h PB treatment. **c**, Functional category (biological process) of 8039 genes with retained introns. Each of these genes has the same intron-retention events in both treatments. The top 20 categories were ordered by the enrichment scores and displayed. **d**, Functional category (biological process) of genes with retained introns only identified in 6-h GEX1A treatment, when compared with 6-h PB treatment. The top 20 categories were ordered by the enrichment scores and displayed

Next, we compared the effects of GEX1A on IR with those of PB. GEX1A generated significantly more IR events (42,649) in a larger number of genes (11,715) compared to PB treatment (21,151 and 8268, respectively). However, we can’t rule out that these differences are due to, at least in part, different doses of the two chemicals and their binding affinities to the SF3B complex in plants. Strikingly, the GEX1A treatment group shared more than 90% of the IR events generated by PB and genes with perturbed splicing and IR (Fig. 7). Functional categorization of the genes with IR events revealed that protein transport, localization, ion homeostasis, spliceosome, protein folding and targeting, and abiotic stresses were the most highly enriched categories, indicating that GEX1A treatment inhibits the splicing machinery and generates splicing stress signals (Fig. 7).

### The effects of GEX1A are partly mediated by ABA signaling

**Fig. 8:**
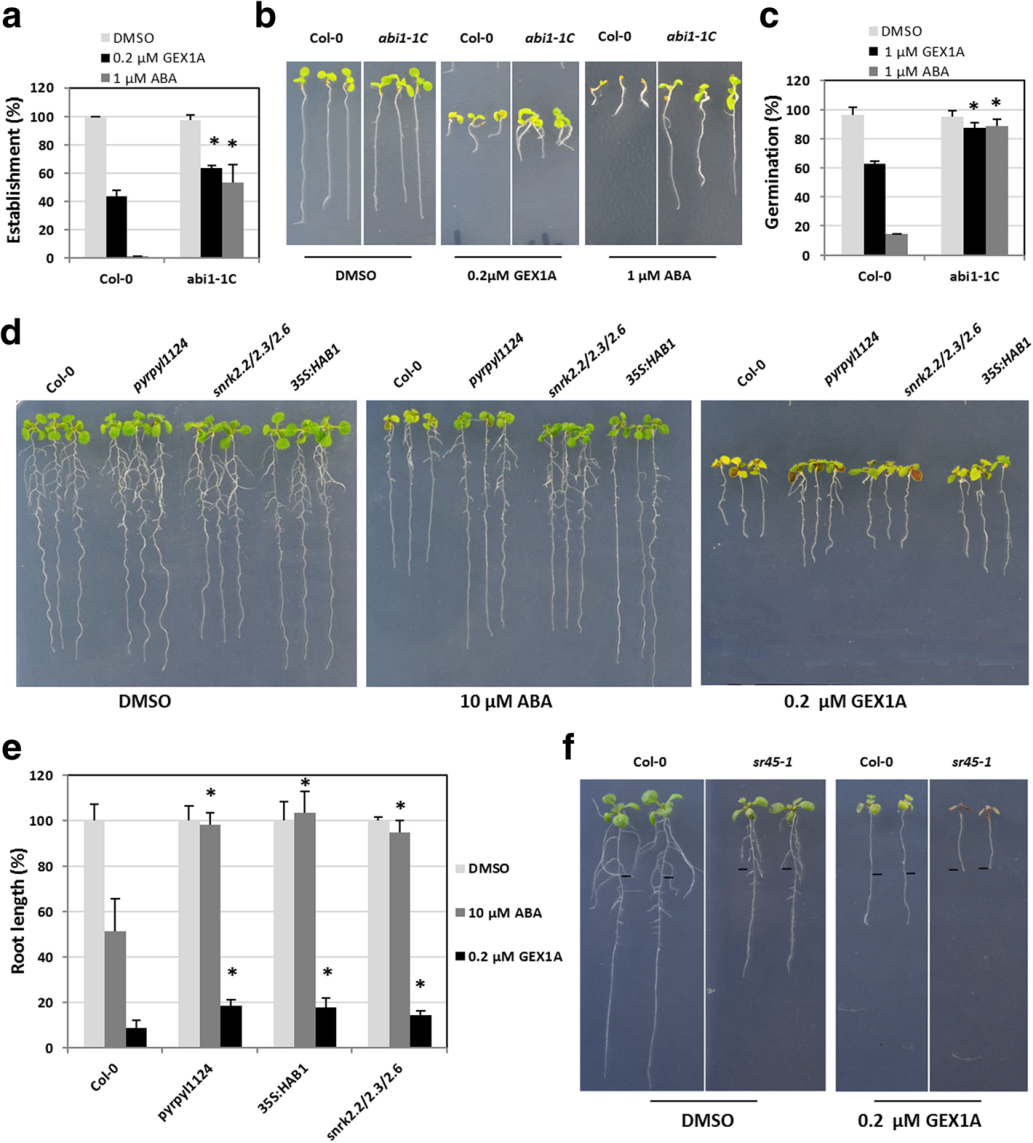
Plants with reduced ABA sensitivity are partially resistant to GEX1A. **a**, The *abi1-1C* mutant is partially resistant to PB in seedling establishment compared to wild-type Col-0 plants. Quantification of seedling establishment (seedlings developing a first pair of true leaves) was performed on MS plates supplemented with DMSO (Control, *white bars*), 0.2 μM GEX1A (*black bars*) or 1 μM ABA (*gray bars*) 8 days after the seeds were sown. Values are averages of 3 independent experiments ± SD (n > 100). * indicates a *p*-value ≤ 0.05 by *t*-test compared to wild type under the same treatment. **b**, Photograph of representative seedlings from **a. c**, The *abi1-1C* mutant is partially resistant to GEX1A in seed germination. Seeds were stratified for 72 h in cold and seed germination (scored as radicle emergence) was calculated 48 h after transfer of the seeds to the controlled growth condition chamber. Values are average of three independent experiments ± SD (n > 100). * indicates a *p*-value ≤ 0.05 by *t*-test compared to wild type under the same treatment. Seeds were sown on MS plates supplemented with DMSO (Control, *white bars*), 1 μM GEX1A (*black bars*) and 1 μM ABA (*gray bars*). **d**, Photograph of representative seedlings showing sensitivities of mutants to ABA and GEX1A. **e**, Plants with reduced sensitivity to ABA are partially resistant to PB in root growth assay. Seedlings grown vertically on MS plates for 3 days were transferred to MS plates containing DMSO (Control, *white bars*), 0.2 μM GEX1A (*black bars*) or 10 μM ABA (*gray bars*). Root length was calculated with ImageJ 7 days after the transfer. Values are average of three independent experiments ± SD (n > 10). * indicates a *p*-value ≤ 0.05 by *t*-test compared to wild type under the same treatment. **f**, 5-day-old Arabidopsis Col (0) wild-type and *sr45-1* mutant seedlings were transferred onto 1⁄2 MS medium with 0.2 μM GEX1A for 4 days. The position of the root tip of seedlings when they were transferred is shown by the *black bars*

Because GEX1A treatment activated ABA-responsive promoters and resulted in transcriptional patterns reminiscent of ABA treatment, we reasoned that the effects of GEX1A could (at least in part) be mediated through the ABA signaling pathway. Therefore, we used different ABA signaling mutants and performed various assays investigating ABA-mediated inhibition of seed germination, seedling establishment, and root growth (Fig. 8). Interestingly, in the seedling establishment assay, after 8 days on medium supplemented with 0.2 μM GEX1A, nearly 63.8% of *abi1-1C* plants produced true leaves compared to less than 43.8% of WT (Col-0) seedlings. Similarly, the *abi1-1C* mutant exhibited 87.4% germination compared to 62.9% of Col-0 seeds on medium supplemented with 1 μM GEX1A. Moreover, *pyrpyl1124*, *snrk2.2/2.3/2.6*, and *35S:HAB1* seedlings grown on 0.2 μM GEX1A exhibited reduced inhibition of root growth compared to Col-0 seedlings. These results indicate that ABA-insensitive mutants and the *35S:HAB1* transgenic line are, to a certain extent, less sensitive to GEX1A than WT. These data are also consistent with our finding for the *sr45* mutant, which is hypersensitive to ABA treatment, i.e., *sr45-1* was more sensitive to low levels of GEX1A treatment than the control (Fig. 8). Specifically, 0.2 μM GEX1A treatment resulted in the complete cessation of *sr45-1* growth, whereas WT seedlings showed reduced but not completely inhibited growth. These results indicate that the effects of GEX1A are mediated, at least in part, through the ABA signaling pathway.

### Differential regulation of splicing of ABA pathway regulators

**Fig. 9:**
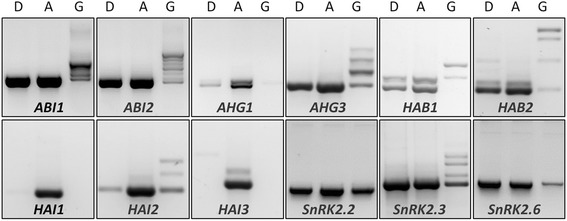
GEX1A affected the splicing of *PP2C* and *SnRK2* genes differently. The cDNAs were prepared from one-week-old Arabidopsis seedlings treated with 5 μM GEX1A or 25 μM ABA for 6 h, with DMSO as control. RT-PCR was performed using primers flanking the first and last exon of each gene. ”D” indicates DMSO treatment, “G” indicates 5 μM GEX1A treatment and “A” indicates 25 μM ABA treatment; gene names are indicated at the bottom of each panel. Functional transcripts of most of PP2C genes were removed by strong intron retention in GEX1A-treated plants, whereas under the same conditions, *SnRK2.2*, *SnRK2.3*, and *SnRK2.6* kept producing functional transcripts with varying levels of intron retention. ABA did not cause obvious intron retention in the selected genes, when compared with DMSO treatment

The above results reveal two obvious, strong effects of GEX1A treatment, i.e., the strong inhibition of splicing with significant accumulation of IR transcripts and the activation of the ABA signaling pathway (and abiotic stress responses in general). To explore the interplay between splicing inhibition and ABA signaling activation, we investigated how the splicing of negative and positive regulators of the ABA pathway is regulated at the level of pre-mRNA splicing. Since GEX1A induces general inhibition of splicing, both negative and positive regulators might be equally inhibited. Conversely, differential splicing regulation might allow more functional transcripts to be produced from positive regulators than from negative regulators, and this titration might lead to the overall activation of ABA signaling. Interestingly, we found that GEX1A treatment led to significant inhibition and accumulation of IR transcripts from PP2Cs, which are negative regulators of the ABA pathway [34–36]. By contrast, GEX1A treatment affected the RNA splicing efficiency of SnRK2 genes, which are positive regulators of the ABA pathway [34, 37, 38], but significant levels of functional transcripts were still produced, indicating that the splicing of positive and negative regulators is differentially regulated. However, we do not entirely exclude other GEX1A effects at the transcriptional levels caused by more complex phenomenon at play, which require further studies (Fig. 9). Furthermore, a PP2C isoform, *HAB1.2* (HYPERSENSITIVE TO ABA1 isoform 2), was recently found to accumulate in response to ABA treatment, resulting in the activation of the ABA pathway [39, 40]. Similarly, this isoform accumulated upon GEX1A treatment, indicating that a conserved, differential splicing regulatory mechanism supports the activation of ABA signaling (Additional file 1: Figure S4). Although our data indicate that the GEX1A treatment led to the activation of the ABA pathway by the regulation of ABA regulators at the post-transcriptional levels. It remains to be tested whether the treatment of GEX1A leads to the induction of ABA synthesis and subsequently the activation of the ABA pathway.

The generation of abiotic stress-like transcription patterns and activation of the ABA signaling pathway in response to GEX1A treatment indicates that even though GEX1A is a general splicing inhibitor, several levels of regulation of differential splicing function to relay the splicing stress signal. To substantiate the presence of differential splicing regulation, we examined the splicing of other splicing factors. For example, splicing factors such as serine/arginine-rich (SR) proteins are positive regulators of splicing and regulate splicing patterns in response to stress cues [41–43]. Moreover, SR genes are themselves alternatively spliced to modulate their functions in pre-mRNA splicing [44]. We therefore investigated whether the splicing patterns of members of the SR/SR-like gene family were affected by GEX1A treatment. Our RNA-seq and RT-PCR data show that these SR/SR-like genes were differentially spliced under GEX1A treatment, with some genes overproducing more functional than nonfunctional (with IR) transcripts. For example, *RS41*, *SR34b*, *SCL28*, and *SC35*, accumulated significant levels of IR transcripts, whereas some other genes, including *SR45*, *SR45a*, *SCL30*, *RSZ32*, produced considerable amounts of constitutive splicing isoforms (functional isoforms) (Additional file 1: Figure S5). These data indicate that despite the inhibition of general splicing by GEX1A, many levels of differential splicing are used to support optimum plant responses to splicing stress signaling, thereby facilitating plant survival.

## Discussion

The regulation of pre-mRNA splicing is a key factor for ensuring that the plant produces the correct transcriptome and proteome, to allow it to respond to different growth and stress cues, thereby ensuring its adaptability and survival [45, 46]. Little is known about the molecular underpinnings of the interplay between the regulation of splicing and abiotic stress responses. Advances in RNA-seq have resulted in the accumulation of vast amounts of transcriptome data for various plant species under a variety of growth and stress conditions [47]. Further analysis of these data will advance our understanding of the molecular adaptation of plants at the transcriptional and post-transcriptional levels [47, 48]. Chemical genetic approaches using small molecules capable of selectively perturbing the splicing machinery are invaluable for understanding the regulation of constitutive and alternative splicing. For example, selective chemical inhibitors can be used in a noninvasive, reversible, tunable manner to probe different layers of the splicing machinery [49].

We recently discovered that PB functions as a selective splicing inhibitor in plant cells [26]. PB, GEX1A, and SSA target the SF3B1 complex [20, 50]. Therefore, we investigated whether GEX1A and SSA exhibit the same or similar physiological and molecular effects. Intriguingly, GEX1A strongly inhibited plant growth and development in a dose-dependent manner. Surprisingly, SSA did not have marked effects on plant growth and development. Moreover, GEX1A exhibited these inhibitory effects on different Arabidopsis ecotypes and different plant species, including rice and tomato, indicating the presence of a conserved molecular target or mechanism. Since GEX1A, like PB, has been implicated in the inhibition of constitutive and alternative splicing, we tested the effects of GEX1A on pre-mRNA splicing of a select group of genes, finding that it indeed inhibited the splicing of genes encoding RNase H, WNK, and NADP-ME2.

To investigate the effects of GEX1A on gene expression and the constitutive and alternative splicing of pre-mRNAs, we performed a genome-wide analysis of its effects on transcriptional and splicing patterns. GEX1A triggered transcriptional patterns similar, to some extent, to those triggered by abiotic stress. For example, GEX1A upregulated genes related to drought and salt stresses. Next, we assessed the effects of GEX1A on pre-mRNA splicing, finding that 53% of intron-containing genes exhibited perturbed splicing. Since GEX1A likely has similar effects on the splicing of genes expressed at low levels, the number of genes whose splicing is perturbed by GEX1A is probably higher. Such strong inhibition of constitutive and alternative splicing by GEX1A indicates that it targets a core complex or sub-complex of the splicing machinery in a manner similar to PB. Since PB had similar effects on the global patterns of gene expression and splicing, with 37% and 25% of intron-containing genes showing perturbed splicing patterns at 6 h and 24 h of treatment, respectively, we compared the genome-wide effects of GEX1A and PB on transcriptional and splicing patterns.

The drastic effect of GEX1A on splicing could be due to the inhibition of a core component of the splicing machinery, resulting in general splicing stress. We investigated the transcriptional patterns triggered by GEX1A-induced splicing stress by performing gene ontology analysis of significantly up- or downregulated genes. Our data show that GEX1A affects the transcriptional patterns of genes related to a variety of abiotic stresses, including abiotic, heat, salt, and drought stress. These findings prompted us to examine the effects of GEX1A on the activation of stress-inducible promoters including the *RD29A* and *MAPKKK18* promoters. These promoters were strongly induced by GEX1A, which mirrors the effect of PB. Because GEX1A activates abiotic stress response genes, we studied its effects on stomatal aperture. GEX1A treatment led to reduced stomatal aperture in a manner similar to that of PB and ABA, indicating that GEX1A activates a stress signal, specifically ABA. Since the SR-related mutant *sr45-1* is hypersensitive to ABA [51, 52], we investigated whether this mutant is hypersensitive to GEX1A. Indeed, the *sr45-1* mutant is hypersensitive to GEX1A. Furthermore, GEX1A treatment led to localization of the SR45:GFP chimeric protein to nuclear speckles, indicating that this drug plays a role in modulation or perturbation of splicing [53].

Constitutive and alternative splicing play major roles in plant growth and adaptation under ever-changing environmental conditions. Since the majority of AS events in plants involve intron retention, one plausible interpretation of the plant responses to different stress and developmental cues is that constitutive and alternative splicing titrate the ratios between functional and non-functional levels of transcripts (isoforms of key regulatory genes). This notion prompted us to examine whether GEX1A treatment would manipulate the ratios of functional versus non-functional transcript levels in the ABA stress response pathway. When plants sense splicing stress, they immediately activate the ABA stress-signaling pathway [38, 54]. Therefore, we tested the effects of GEX1A on the production of functional and non-functional levels of transcripts of negative and positive regulators of the ABA pathway. SnRK2s (positive regulators) maintained a substantial fraction of functional transcripts compared to PP2Cs (negative regulators), which had much higher levels of nonfunctional transcripts. This result indicates that splicing stress signaling triggered by GEX1A activates the ABA pathway by modulating the levels of functional and nonfunctional transcripts of the regulators of this pathway. Splicing regulation is an important mechanism that helps plants adapt and respond to stress conditions. SR proteins are key regulators of CS and AS; these proteins regulate various aspects of RNA metabolism, including splicing, processing, export, and translation. We therefore studied the effects of GEX1A on the transcriptional and splicing patterns of SR proteins. We found that SR genes are differentially spliced, indicating that splicing stress signaling triggered by GEX1A treatment results in differential splicing regulation of pre-mRNAs, most likely to help ensure plant survival.

We found that PB and GEX1A have similar effects on gene expression and splicing patterns. Furthermore, these drugs are strong inhibitors of constitutive and alternative splicing, indicating that they target the same molecular component or complex. A recent study showed that although the splicing inhibitors PB, GEX1A, and SSA are structurally unrelated, they share the same molecular target in mammalian systems [20]. Interestingly, SSA does not have tangible effects on plant growth and development. Therefore, the use of these compounds in plants could help uncover the molecular roles of U2snRNP (and its sub-complexes) in splicing regulation and its impact on transcriptional patterns under a variety of growth and stress conditions. Plants are excellent systems for elucidating the molecular roles of splicing inhibitors. Such investigations would advance our understanding of splicing in plants and eukaryotes in general. In addition, these inhibitors could potentially be used by clinicians as targeted therapeutic compounds to treat diseases. Therefore, small molecule splicing inhibitors could be used to uncover the molecular underpinnings of the splicing process and its interacting regulatory networks. Substantial evidence connects the regulation of splicing to a variety of developmental and stress responses. Such small molecular inhibitors could be used to probe the interplay between splicing regulation and various growth, developmental, and stress cues.

Our work highlights the interconnectedness between the splicing machinery and stress responses, thereby linking stress signaling to the ABA pathway at the post-transcriptional level of regulation. Our results show that 1) GEX1A is a strong inhibitor of plant growth and development and an inhibitor of constitutive and alternative splicing; 2) GEX1A induces stress-related transcriptional patterns similar to those triggered by PB, indicating that these drugs share the same molecular target; 3) GEX1A activates the ABA pathway and ABA-induced stress promoters: GEX1A activates abiotic stress response genes and leads to stomatal closure; and 4) GEX1A activates the ABA pathway by modulating the splicing of positive and negative regulators of this pathway.

## Conclusions

Our study establishes GEX1A as a splicing inhibitor and modulator and indicates that GEX1A and PB target the same component of the spliceosome machinery. GEX1A and PB can be used to explore the post-transcriptional regulation of stress responses and the interplay between splicing stress and abiotic stress conditions. Furthermore, our study points to the validity of screening chemical libraries for splicing inhibitors using plant systems, developing splicing inhibitors for potential use as herbicides, and engineering plants with resistance against these splicing inhibitors.

## Methods

### Plant materials and growth conditions

Seeds of wild-type *Arabidopsis thaliana* Col-0, wild-type L*er*, *RD29A::LUC* (the firefly luciferase reporter gene under the control of the stress-responsive RD29A promoter, C24 background [55]), *35S::SR45.1:GFP* [56]*, 35S::HAB1*( HAB1, HYPERSENSITIVE TO ABA1, [57])*, MAPKKK18::uidA (*reporter gene *uidA* driven by *MAPKKKK18* promoter)*,* and the *pyrpyl1124* (quadruple mutants of *PYR1*, *PYL1*, *PYL2* and *PYL4* genes, [35])*, abi1-1C* (*abi1*, a ABA insensitive mutant in Col-0 background [58]), *snrk2.2/2.3/2.6* (triple mutants of SnRK2 genes*,* [37]) and *sr45-1* mutants [51] were surface sterilized with 10% bleach for 10 min and used directly for seed germination assays or stored at 4 °C for 2 days. The seeds were plated on ½ × Murashige and Skoog (MS) medium agar plates supplemented with 1% sucrose and the indicated chemicals. The plates were placed in a growth chamber (Model CU36-L5, Percival Scientific, Perry, IA, USA) under 16 h-white light (~75 μmol m^−2^ s^−1^) and 8 h-dark conditions at 22 °C for germination and seedling growth.

### Chemicals

GEX1A (CAS: 142861-00-5) was purchased from BOC Sciences (45-16 Ramsey Road, Shirley, NY 11967, USA). Spliceostatin A (CAS: 391611-36-2) was purchased from Adooq Bioscience (Irwin, CA, USA).

### RNA extraction and RNA-seq

Total RNA was extracted from seedlings after the indicated treatments (DMSO and 5 μM GEX1A) for 6 h using TRIzol Reagent (Catalog No. 15596–026, Invitrogen). Polyadenylated RNA was isolated using an Oligotex mRNA Midi Kit (70042, Qiagen Inc., Valencia, CA, USA). The RNA-seq libraries were constructed using an Illumina Whole Transcriptome Analysis Kit following the standard protocol (Illumina, HiSeq system) and sequenced on the HiSeq platform to generate high-quality paired-end reads.

### Analysis of RNA-seq data and gene functional classification

The annotated Arabidopsis gene models were downloaded from TAIR10 (https://www.arabidopsis.org/). TopHat (Version 2.0.10) was used for alignment and to predict splice junctions [59]. Gene expression levels (FPKM values) were calculated using Cufflinks (Version 2.0.0). The DEGs were identified using Cufflink and the limma package in R. Very strict criteria were used to define DEGs: DEGs must simultaneously show more than 1.8-fold upregulation/downregulation in both replicates, and *p*-values calculated by limma must be less than 0.05. To filter out false positive junctions, well-studied criteria (i.e., an overhang size of more than 20 bp and at least two reads spanning the junctions) were set as cutoff values [60]. JuncBASE was used to annotate all AS events based on the input genome coordinates of all annotated exons and all confidently identified splice junctions [61]. Fisher’s Exact Tests were used to identify differential representation of each type of AS event. For intron retention, Fisher’s Exact Tests were performed on the intron-read counts and the corresponding exon-read counts between control and 6 h drug treatment. Events with *p*-value < 0.001 were identified as significantly different. In addition, intron retentions uniquely identified in the control or treatment groups were considered significant if there was at least five-fold coverage of support and the p-values of these events were assigned to zero. For alternative 5'SSs and 3'SSs and exon skipping events, Fisher’s Exact Tests were performed on the comparisons of the junction-read counts and the corresponding exon-read counts between the control and 6 h drug treatment. Events with p-values less than 0.05 were identified as significantly different. GO classifications were performed with DAVID software. GO network analysis was performed with EGAN.

### RT-PCR and RT-qPCR

For reverse-transcription quantitative PCR (RT-qPCR), DNA digestion of total RNA samples was performed after RNA extraction using an RNase-Free DNase Set (Invitrogen cat. No. 18068-015) following the manufacturer’s protocol. The total RNA was reverse transcribed using a SuperScript First-Strand Synthesis System for RT-qPCR (Invitrogen) to generate cDNA. Primers used for RT-PCR are listed in Additional file 2: Table S1.

### Germination rate assay

Freshly harvested Arabidopsis Col-0 seeds were surface sterilized, plated on control or chemical-containing MS agar plates, incubated in a 22 °C growth chamber, and photographed at the indicated time points under a stereomicroscope (Nikon, SMZ 25). According to Piskurewicz et al., seeds with radicle lengths that reach 1/3 of the seed length were scored as germinated [62].

### *RD29A::LUC* analysis

Intact 10-day-old *RD29A::LUC* plants were treated with 0.05% DMSO, 5 μM GEX1A, or 100 μM ABA for 5–6 h and sprayed with 1 mM D-luciferin (Gold Biotechnology, St. Louis, MO, USA). The plates were incubated for 5 min in the dark before luminescence imaging under a CCD camera (ANDOR).

### Stomatal aperture assays

Rosette leaves from 2–3-week-old plants were floated in a solution containing 50 μM CaCl_2_, 10 mM KCl, and 10 mM MES-Tris (pH 6.15) and exposed to light (150 μmol m^-2^ sec^-l^) for at least 2.5 h. Subsequently, 20 μM DMSO, GEX1A, or ABA was added to the solution to assay for stomatal closure [63]. After treatment for 4 h, stomatal apertures in plant tissue on a microscope slide were photographed immediately under a light microscope (Carl Zeiss, Axio Imager.2) at 400× magnification. After image acquisition, the stomatal apertures were measured with the open access software Image J (Version 1.37) as previously described [64].

### Subcellular localization of SR45 protein

Five-day-old *35S::SR45.1-GFP* transgenic seedlings were incubated in 0.01% DMSO with 5 μM GEX1A for 6 h and viewed under a Zeiss laser-scanning microscope (Carl Zeiss Meta 710, Wetzlar, Germany) with a 488-nm argon laser and a long-pass 530 filter. Serial optical sections were collected and projected with Zeiss LSM Image Browser software (Carl Zeiss) and Photoshop version 7.0 software (Adobe).

## Additional files

Additional file 1: This file contains all supporting Supplementary Figures. (PDF 861 kb)

Additional file 2: Table S1. includes information for primers used in this paper. (XLSX 14 kb)

## Abbreviations

5′SS: 5′ splice site
ABA: Abscisic acid
AS: Alternative splicing
BPS: branch point sequence
DAS: days after sowing
DEG: Differentially expressed gene
EGAN: Exploratory gene association networks
FPKM: Fragments per kilobase of transcript per million mapped reads
GEX1A: Herboxidiene
IR: intron retention
MS: Murashige and Skoog
PB: Pladienolide B
RNA-seq: RNA-sequencing
RT-qPCR: reverse-transcription quantitative PCR
SR: serine/arginine-rich
SSA: Spliceostatin A
