# Supplementary material for: Herboxidiene triggers splicing repression and abiotic stress responses in plants
Source: BMC Genomics. 2017 Mar 27;18:260. doi: 10.1186/s12864-017-3656-z (PMC5369228; doi:10.1186/s12864-017-3656-z)
Supplement: Supplementary file 1 — This file contains all supporting Supplementary Figures. (PDF 861 kb) [file 12864_2017_3656_MOESM1_ESM.pdf]

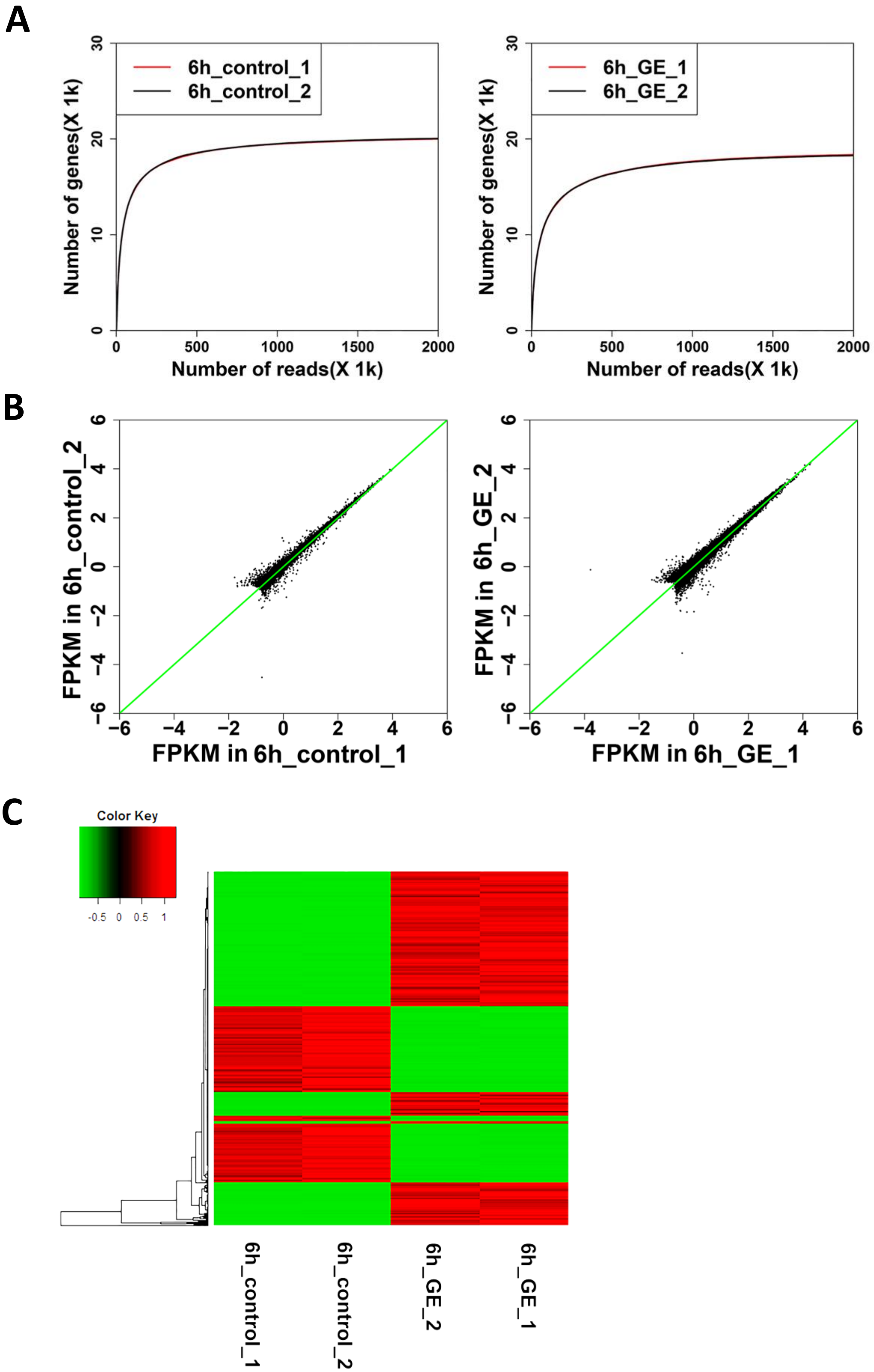

Supplementary Figure 1. High quality of RNA-seq data. A, Saturation curve for gene detection. Randomly sampled reads were plotted against the expressed genes. **B**, Comparison of gene expression between the two replicates. The FPKM values were plotted. **C**, the clustering of gene expression levels between control and GEX1 treatments. The figure shows a consistency between the two replicates, 408 genes were down regulated and 561 genes were up regulated by GEX1A treatment.

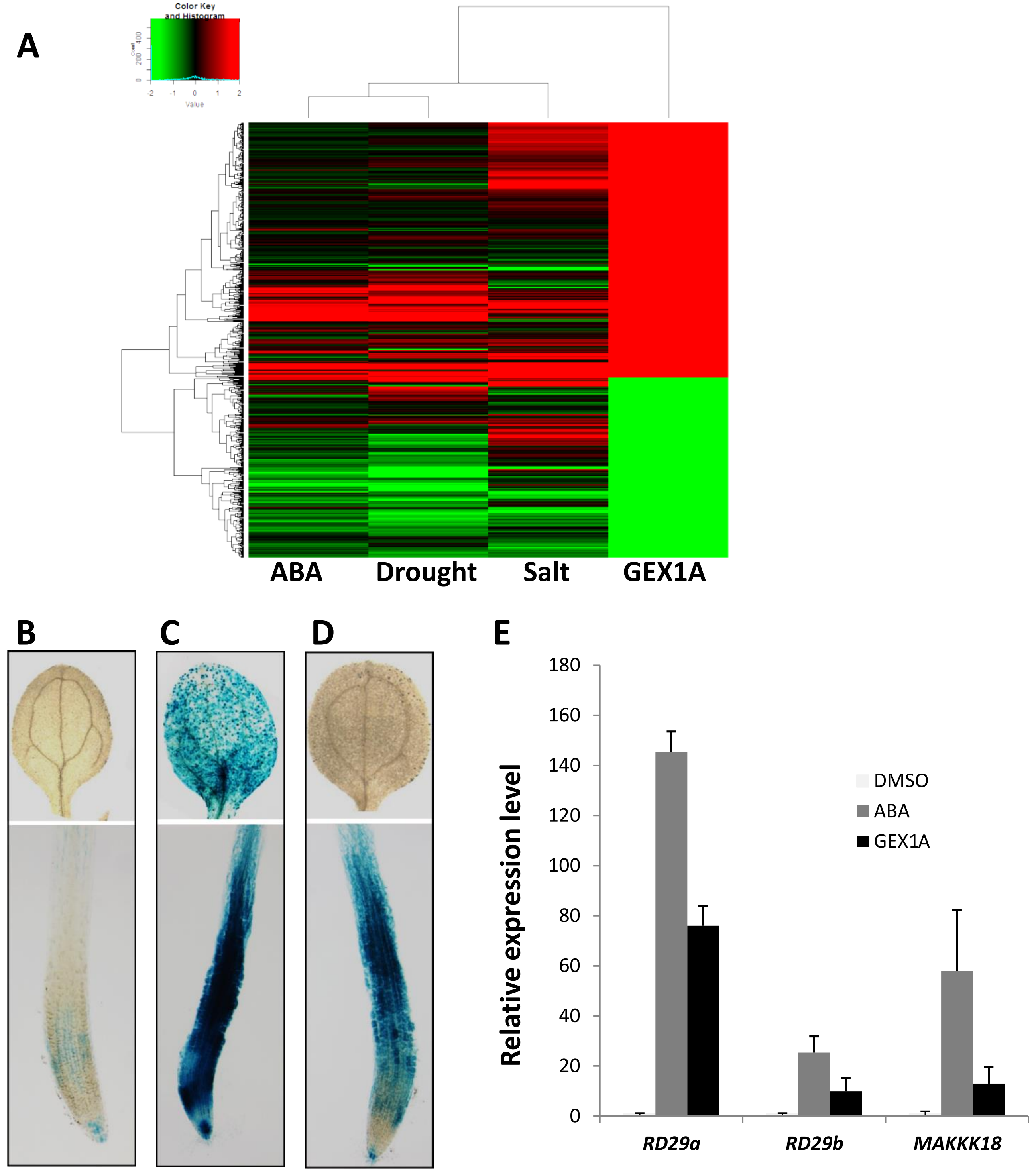

Supplementary Figure 2. GEX1A induce stresses/ABA responsive gene expression. A, A heatmap was generated by mapping upregulated and downregulated ($\log_2$(fold_change)≥2) genes in GEX1A treatment to gene expression profiles from studies on ABA, drought and salt treatment (microarray database using Genevestigator). The heatmap indicates that a number of GEX1A upregulated genes are upregulated by ABA, drought and salt stress (red); and similarly, a number of GEX1A downregulated genes are downregulated by ABA, drought and salt stress (green).. **B-D,** GEX1 induced *MAPKKK18:GUS* reporter gene expression, 10 day old *MAPKKK18:GUS* reporter transgenic plants were incubated in 20 µM GEX1A for 6h, followed by GUS staining. The GEX1A treated plants (**D**) showed stronger GUS signal in the root, when compared with negative control (**B**), ABA was used as positive control. **E**, quantative RT-PCR showed PB inducing endogenous *RD29a*, *RD29b* and *MAKKK18* highly expression. The cDNAs were prepared from one week old Arabidopsis Col-0 wild type seedlings previously treated by 5 µM GEX1A and 25 µM ABA for 6h respectively, DMSO as control.

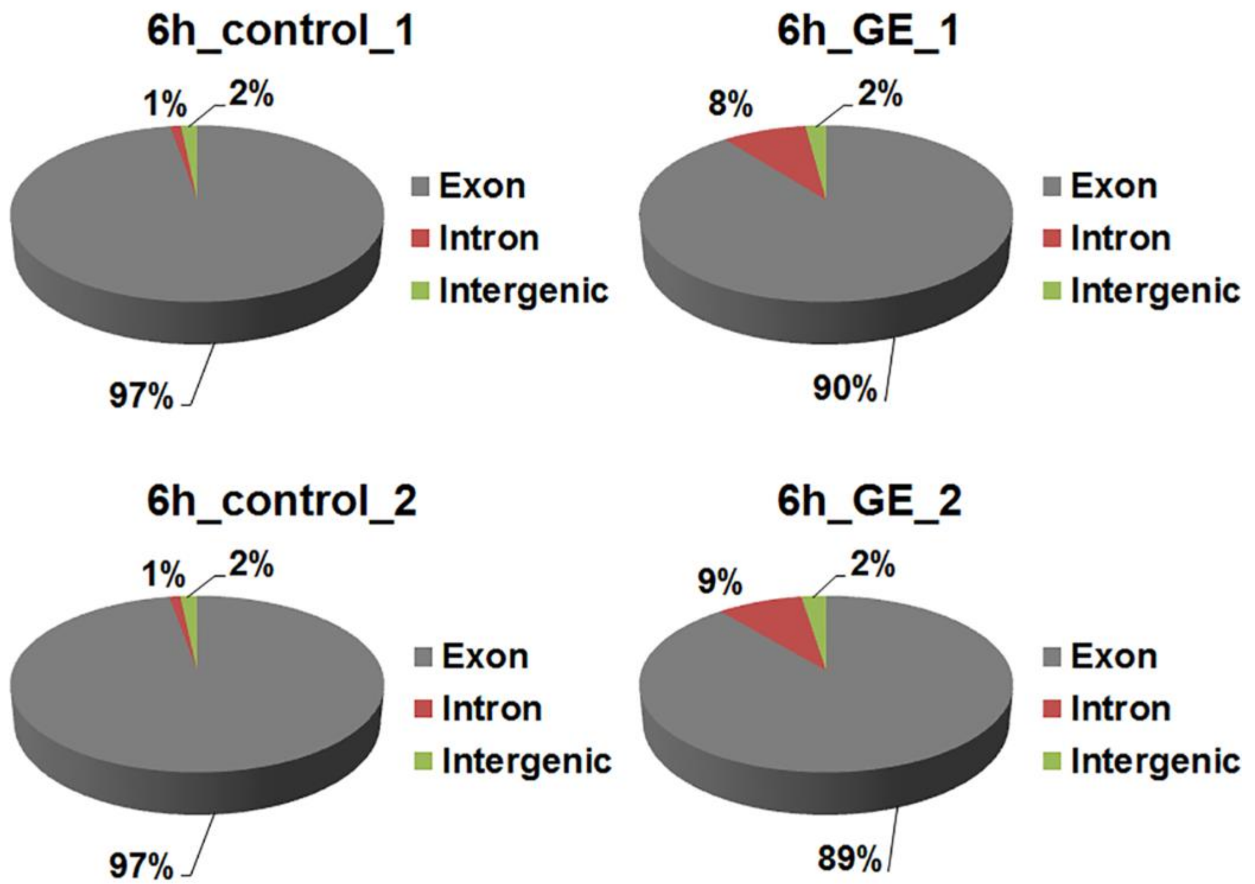

Supplementary Figure 3. Distribution of the RNA-seq reads along annotated Arabidopsis genomic features.

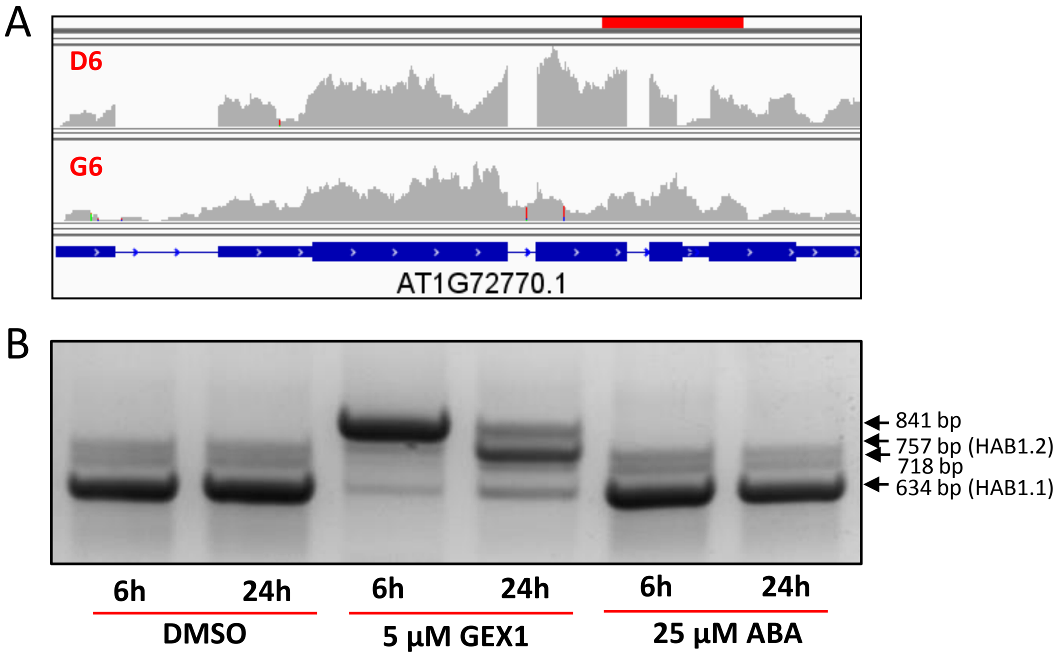

Supplementary Figure 4. GEX1 treatment induced high expression of the *HAB1.2* isoform. A, snapshot of IGV for *HAB1* (AT1G72770) from RNA-seq data, showing the gene structure and splicing pattern in 5 µM GEX1A treatment for 6 h, DMSO as control. **B**, RT-PCR. The cDNAs were prepared from one-week-old Arabidopsis seedlings, which were treated with 5 µM GEX1A or 25 µM ABA, 0.5% DMSO for 6 h and 24 h, respectively. RT-PCR demonstrated that the *HAB1.2* variant is the major isoform after 5 µM GEX1A treatment for 24 h. The 634-bp band was considered as the *HAB1.1* variant, and the 757-bp band was the *HAB1.2* variant.

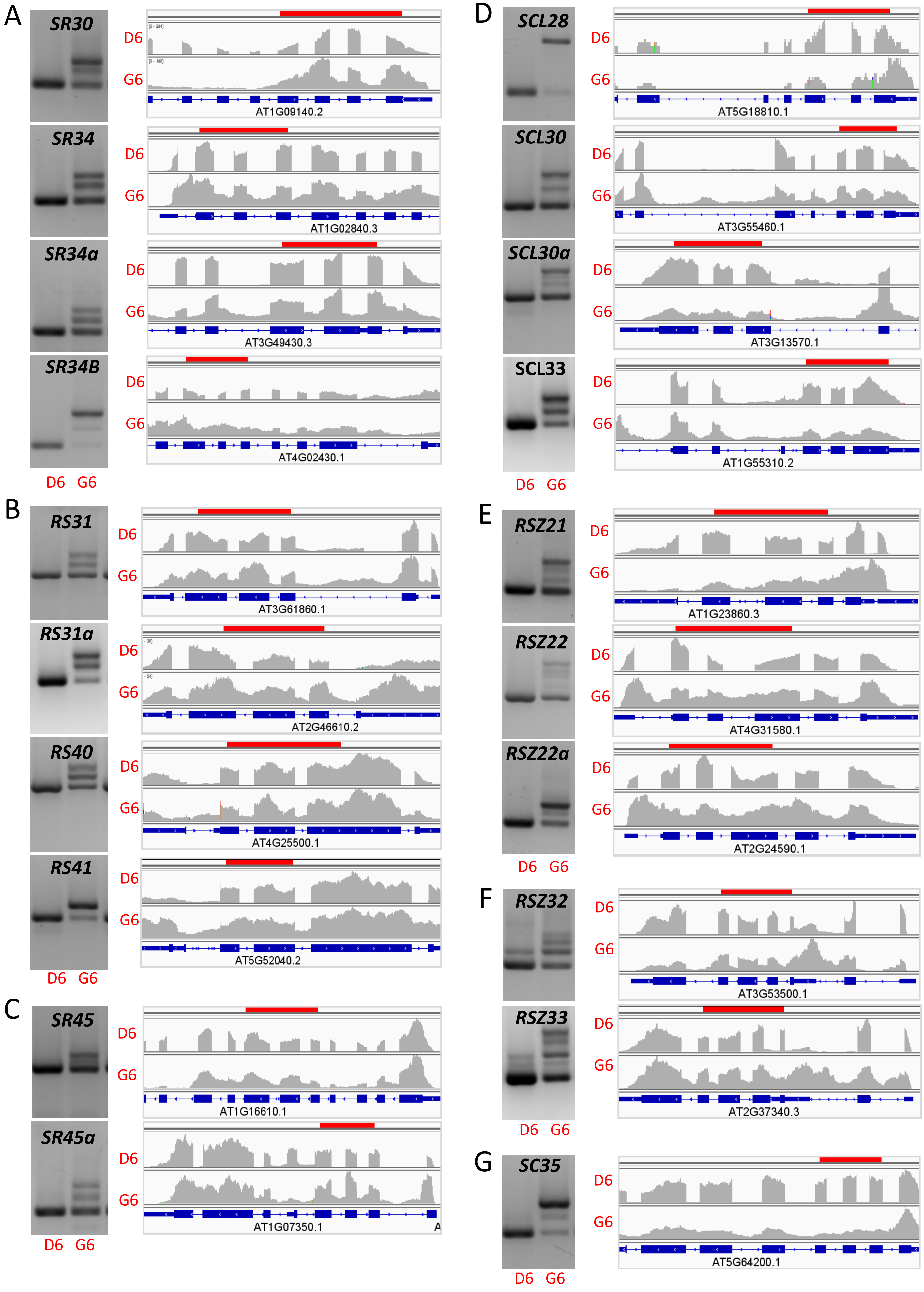

A
SR30
AT1G09140.2
SR34
AT1G02840.3
SR34a
AT3G49430.3
SR34B
AT4G02430.1
B
RS31
AT3G61860.1
RS31a
AT2G46610.2
RS40
AT4G25500.1
RS41
AT5G52040.2
C
SR45
AT1G16610.1
SR45a
AT1G07350.1
D
SCL28
AT5G18810.1
SCL30
AT3G55460.1
SCL30a
AT3G13570.1
SCL33
AT1G55310.2
E
RSZ21
AT1G23860.3
RSZ22
AT4G31580.1
RSZ22a
AT2G24590.1
F
RSZ32
AT3G53500.1
RSZ33
AT2G37340.3
G
SC35
AT5G64200.1
D6
G6

Supplementary Figure 5. GEX1A-induced intron retention in SR and SR- like subfamily proteins. The cDNAs were prepared from one-week-old Arabidopsis seedlings treated with 5 µM GEX1A for 6 h, with DMSO as control. IGV snapshot and validation of the intron retention in SR/SR-like genes by RT-PCR using intron-flanking primers. **A**, SR subfamily genes *SR30*, *SR34*, *SR34a*, and *SR34b*. **B**, RS subfamily genes *RS31*, *RS31a*, *RS40*, and *RS41*. **C**, SR-like subfamily genes *SR45* and *SR45a*. **D**, SCL subfamily genes *SCL28*, *SCL33*, *SCL30*, and *SCL30a*. **E**, RSZ subfamily genes, including *RSZ21*, *RSZ22*, and *RSZ22a* underwent intron retention after PB treatment. **F**, RS2Z subfamily genes *RS2Z32* and *RS2Z33* underwent intron retention after PB treatment. **G**, the SC subfamily gene *SC35*. D6, 6-h DMSO treatment; G6, GEX1A 6-h treatment.
